# A new *ex vivo* method to evaluate the performance of candidate MRI contrast agents: a proof-of-concept study

**DOI:** 10.1186/1477-3155-12-12

**Published:** 2014-04-05

**Authors:** Ana Paula Candiota, Milena Acosta, Rui Vasco Simões, Teresa Delgado-Goñi, Silvia Lope-Piedrafita, Ainhoa Irure, Marco Marradi, Oscar Bomati-Miguel, Nuria Miguel-Sancho, Ibane Abasolo, Simó Schwartz, Jesús Santamaria, Soledad Penadés, Carles Arús

**Affiliations:** 1 Centro de Investigación Biomédica en Red – Bioingeniería, Biomateriales y Nanomedicina (CIBER-BBN), Spain; 2 Departament de Bioquímica i Biologia Molecular, Unitat de Bioquímica de Biociències, Edifici Cs, Universitat Autònoma de Barcelona, Cerdanyola del Vallès, Barcelona 08193 Spain; 3 Institut de Biotecnologia i de Biomedicina, Universitat Autònoma de Barcelona, Cerdanyola del Vallès, Barcelona 08193 Spain; 4 Servei de RMN, Universitat Autònoma de Barcelona, Edifici C, Cerdanyola del Vallès, Barcelona 08193 Spain; 5 Centro de Investigación Cooperativa en Biomateriales - CIC biomaGune, Pª Miramón182, San Sebastián 20009 Spain; 6 Departamento de Física Aplicada, Facultad de Ciencias, Universidad Autónoma de Madrid, Cantoblanco Madrid 28049 Spain; 7 Instituto de Investigación en Nanociencia de Aragón (INA), Edificio Interfacultades II. C/ Pedro Cerbuna, 12. Universidad de Zaragoza, Zaragoza 50009 Spain; 8 CIBBIM-Nanomedicine, Vall d’Hebron Institut de Recerca (VHIR), Hospital Universitari Vall d’Hebron, Universitat Autònoma de Barcelona, Barcelona 08035 Spain

**Keywords:** Magnetic nanoparticles, Magnetic resonance imaging, Contrast media, *ex vivo* screening

## Abstract

**Background:**

Magnetic resonance imaging (MRI) plays an important role in tumor detection/diagnosis. The use of exogenous contrast agents (CAs) helps to improve the discrimination between lesion and neighbouring tissue, but most of the currently available CAs are non-specific. Assessing the performance of new, selective CAs requires exhaustive assays and large amounts of material. Accordingly, in a preliminary screening of new CAs, it is important to choose candidate compounds with good potential for *in vivo* efficiency. This screening method should reproduce as close as possible the in vivo environment. In this sense, a fast and reliable method to select the best candidate CAs for *in vivo* studies would minimize time and investment cost, and would benefit the development of better CAs.

**Results:**

The post-mortem *ex vivo* relative contrast enhancement (RCE) was evaluated as a method to screen different types of CAs, including paramagnetic and superparamagnetic agents. In detail, sugar/gadolinium-loaded gold nanoparticles (Gd-GNPs) and iron nanoparticles (SPIONs) were tested. Our results indicate that the post-mortem e*x vivo* RCE of evaluated CAs, did not correlate well with their respective *in vitro* relaxivities. The results obtained with different Gd-GNPs suggest that the linker length of the sugar conjugate could modulate the interactions with cellular receptors and therefore the relaxivity value. A paramagnetic CA (GNP (E_2)), which performed best among a series of Gd-GNPs, was evaluated both *ex vivo* and *in vivo*. The *ex vivo* RCE was slightly worst than gadoterate meglumine (201.9 ± 9.3% versus 237 ± 14%, respectively), while the *in vivo* RCE, measured at the time-to-maximum enhancement for both compounds, pointed to GNP E_2 being a better CA in vivo than gadoterate meglumine. This is suggested to be related to the nanoparticule characteristics of the evaluated GNP.

**Conclusion:**

We have developed a simple, cost-effective relatively high-throughput method for selecting CAs for *in vivo* experiments. This method requires approximately 800 times less quantity of material than the amount used for *in vivo* administrations.

## Background

Magnetic resonance imaging (MRI) plays an important role in early tumor detection and diagnosis. The use of exogenous contrast agents (CAs) [1,2] is helpful to improve the discrimination between lesion and neighbouring tissue.

Contrast agents (CAs) can enhance tissue contrast by shortening longitudinal (T_1_) and transverse (T_2_) relaxation times of water. The magnitude of this effect is measured as relaxivity (r_1_ or r_2_) and it is related to the efficacy of the CAs in accelerating relaxation. This parameter can be modulated by many factors, such as protein binding, chemical structure and dynamic processes (e.g. water exchange kinetics, first and second coordination sphere hydration, rotational tumbling rates) [3,4]. When administered at equivalent doses, compounds with high relaxivity values could generate greater contrast than compounds with lower relaxivity, and therefore less amount is required to generate the same effect. On the other hand, compounds with low *in vitro* relaxivity but with specific binding to some cellular/molecular targets can also have potential application as CAs in MRI [5]. Most of the currently available extracellular CAs are non-specific and less efficient than predicted by theory [6], and most of the *in vitro* strategies used to evaluate their performance are not able to reproduce the *in vivo* conditions that could modify their ability to generate contrast [5,7-9].

Analyzing the *in vivo* performance of a new candidate CA involves exhaustive assays and requires large amounts of material, not always available at early stages of product development. Accordingly, a preliminary screening of new CAs should ideally choose candidates with good *in vivo* potential, in order to minimize time and cost invested. In this process, one also needs to consider the ability of the CA to reach the target tissue and to interact with the extracellular matrix or substances released by cells. In this sense, the standard *in vitro* method currently used for evaluating CA performance, relaxivity calculation [10], may not be a good predictor of *in vivo* performance. Up to date, there is not any “ideal” *ex vivo* method, although some have been described [11,12]. The use of a fast and reliable method to select CAs from a range of candidates, reproducing as close as possible the biological environment and requiring small amounts of material, would benefit the development of new and better CAs.

**Figure 1:**
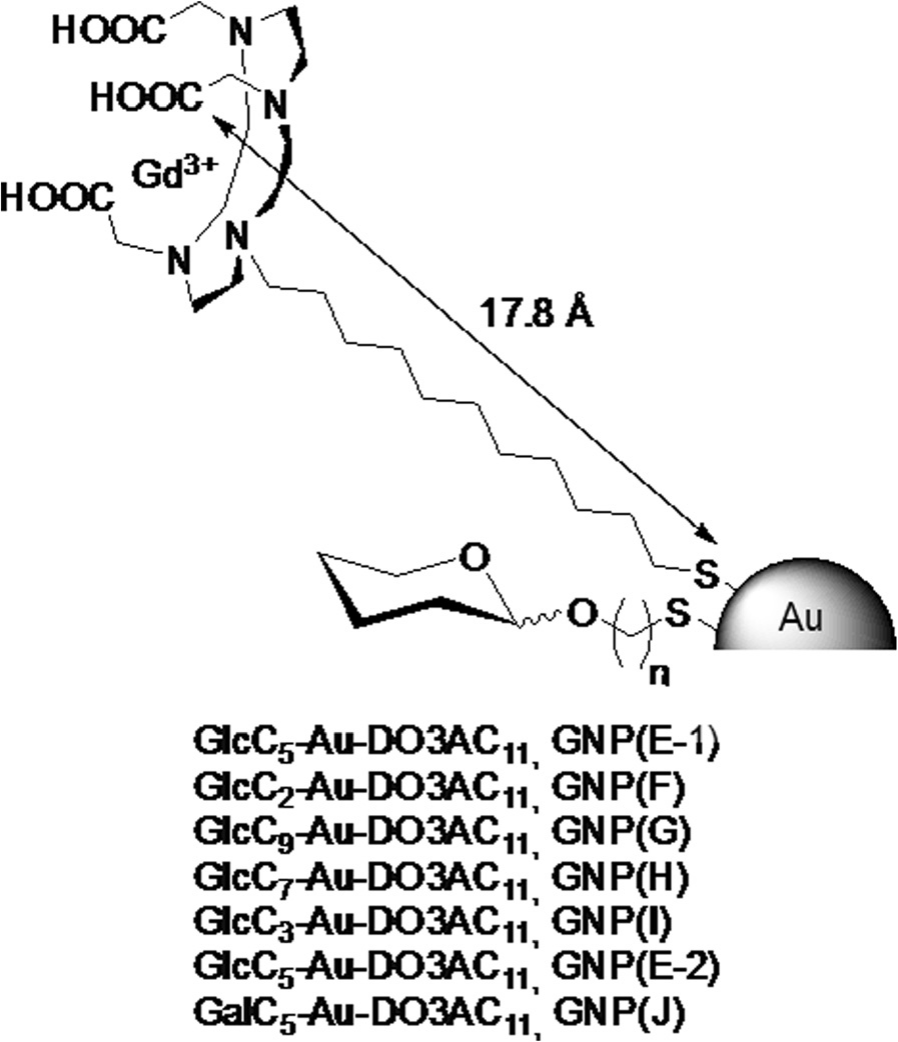
**Short name: Schematic representation of the paramagnetic Gd**-**chelate containing gold glyconanoparticle (Gd**-**GNPs).** Detailed legend: Schematic representation of the paramagnetic Gd-chelate containing gold glyconanoparticle (Gd-GNPs) tested in this work. The gold core (Au) is coated with Gd:DO3A derivatives and self-assembled monolayer of saccharide conjugates. Thiol-ended alkane linkers are used as spacers to attach the conjugates to the gold core. Figure not drawn to scale. Glc = glucose. See also Table 1, text and reference [7] for further details.

The purpose of this work was to design and evaluate a simple *ex vivo* method to assess the performance of new CAs for preclinical brain tumor detection using minimal amounts of material. This method should be a better predictor of *in vivo* performance than classic *in vitro* relaxivity measurements. To validate it, we have tested novel paramagnetic Gd-based gold nanoparticles (Gd-GNPs) (Figure 1) [7,13] as well as superparamagnetic iron oxide nanoparticles (SPIONs) [14]. The Gd-GNPs incorporate several copies of sugars (which confer biocompatibility and water solubility) and Gd complexes. The multivalent presentation of sugars in these Gd-GNPs gives them targeting properties, enhancing their avidity for carbohydrate-binding receptors at the cell surface and could also modulate their cellular uptake.

Iron oxide, and specifically iron oxide nanoparticles, are the most investigated material for negative CA in biomedicine because of its high magnetic moment, chemical stability in physiological conditions and low toxicity [15,16]. Further engineering of these nanoparticles should improve their performance, making them more selective and thus more efficient [17-20]. Nevertheless, some of the modifications introduced in the iron nanoparticles can alter their superparamagnetic properties (and, hence, the contrast effect obtained) which should be evaluated before going further in nanoparticle development.

## Results

Post mortem *ex vivo* RCE analysis and *in vitro* 1.4 T and 7.0 T relaxivity measurements were performed for all nanoparticles, while *in vivo* measurements in mice were restricted to the best performing Gd-GNP candidate.

### *In vitro* relaxivity studies

The r_1_ relaxivity values were measured for positive contrast agents, both at 1.4 and 7.0 T, and are listed in Table 1. As expected, r_1_ decreases with increasing magnetic field [21]. At 1.4 T, all the Gd-GNPs relaxivity values were higher than gadoterate meglumine, whereas at 7.0 T a higher dispersion was observed, with values both above and below gadoterate meglumine. It should be noted that the value obtained *in vitro* at 7.0 T for gadoterate meglumine (one of the gold standards for Gd-containing CAs), is in good agreement with literature values obtained *in vivo* for Gd-DTPA at 6.3 T (3.0 mM^−1^ s^−1^) [22], although at slightly different temperature (23°C *in vitro* and 37°C *in vivo*).

**Table 1:** **Gd**-**GNPs studied**, **percentage of Gd (in g of Gd per 100 g of nanoparticle), relaxivity values (r**_**1**_**, r**_**2**_**and r**_**2**_**/r**_**1**_**) and relative contrast enhancement (RCE) values**

**Contrast agent Gd**-**GNP (Glyco**-**Au**-**D03A:Gd)**^**a**^	**% Gd (g of Gd per 100 g of nano particle)**	**1.4 T r**_**1**_**relaxivity (s**^−**1**^ **mM**^−**1**^**) (in vitro) (n = 3)**	**7.0 T r**_**1**_**relaxivity (s**^**1**^ **mM**^−**1**^**) (in vitro) (n** = **3)**	**7.0 T r**_**2**_**relaxivity (s**^−**1**^ **mM**^−**1**^**) (in vitro) (n** = **3)**	**7.0 T r**_**2**_/**r**_**1**_	**% RCE postmorten (ex vivo) (n** = **3)**	% RCE (in vivo**) (n = **3)**
GlcC5-DO3AC11 (GNP (E_1))	4.7 ± 0.1	7.4 ± 0.7	2.7 ± 0.40	20.2 ± 0.11	7.48	182.8 ± 10.7*	NM
GlcC2-DO3AC11 (GNP (F))	3.4 ± 0.2	7.1 ± 0.9	2.9 ± 0.04	14.5 ± 2.37	5.00	185.4 ± 17.2	NM
GlcC9-DO3AC11 (GNP (G))	3.2 ± 0.2	7.5 ± 0.6	1.6 ± 0.15	12.7 ± 0.24	7.94	168.7 ± 7.74*	NM
GlcC7-DO3AC11 (GNP (H))	4.1 ± 0.2	7.1 ± 0.9	1.6 ± 0.36	11.2 ± 0.21	7.00	143.6 ± 8.0*	NM
GlcC3-DO3AC11 (GNP (I))	3.3 ± 0.2	6.3 ± 0.7	1.8 ± 0.07	19.9 ± 1.43	11.06	141.5 ± 2.5	NM
GlcC5-DO3AC11 (GNP (E_2))^b^	7.0 ± 0.2	11.5 ± 0.1	8.2 ± 0.37	14.5 ± 0.66	1.77	201.9 ± 9.3*	124.9 ± 8.3*
GlcC5-DO3AC11 (GNP (J))	5.0 ± 0.2	8.1 ± 0.3	4.6 ± 0.24	24.4 ± 0.34	5.30	169.1 ± 14.1*	NM
Gadoterate meglumine	NA	3.10^c^/3.5^d^	2.1 ± 0.42	3.58 ± 0.02	1.70	236.7 ± 8.3	113. 1 ± 2.5

Detailed legend:

^a^)Glc = glucose, Gal = galactose. C (number) after the saccharide and the DO3A indicates the number of carbon atoms of the linkers.

^b^) GNP (E_2) was obtained from GNP (E_1) after further incubation with a solution of DO3AC11-Gd complex (4 equiv.) during 44 hours as described in [31].

^c^) Values taken from [7].

^d^) Values taken from [58].

*p < 0.05 versus gadoterate meglumine.

**Mean of RCE values (n = 9 independent measures for 3 animals) measured at the maximum enhancement time, for each of the CA studied *in vivo*.

*NM*, not measured. NA, not applicable.

For each value mean and standard deviation are shown.

### *Ex vivo* post mortem MRI analysis

**Figure 2:**
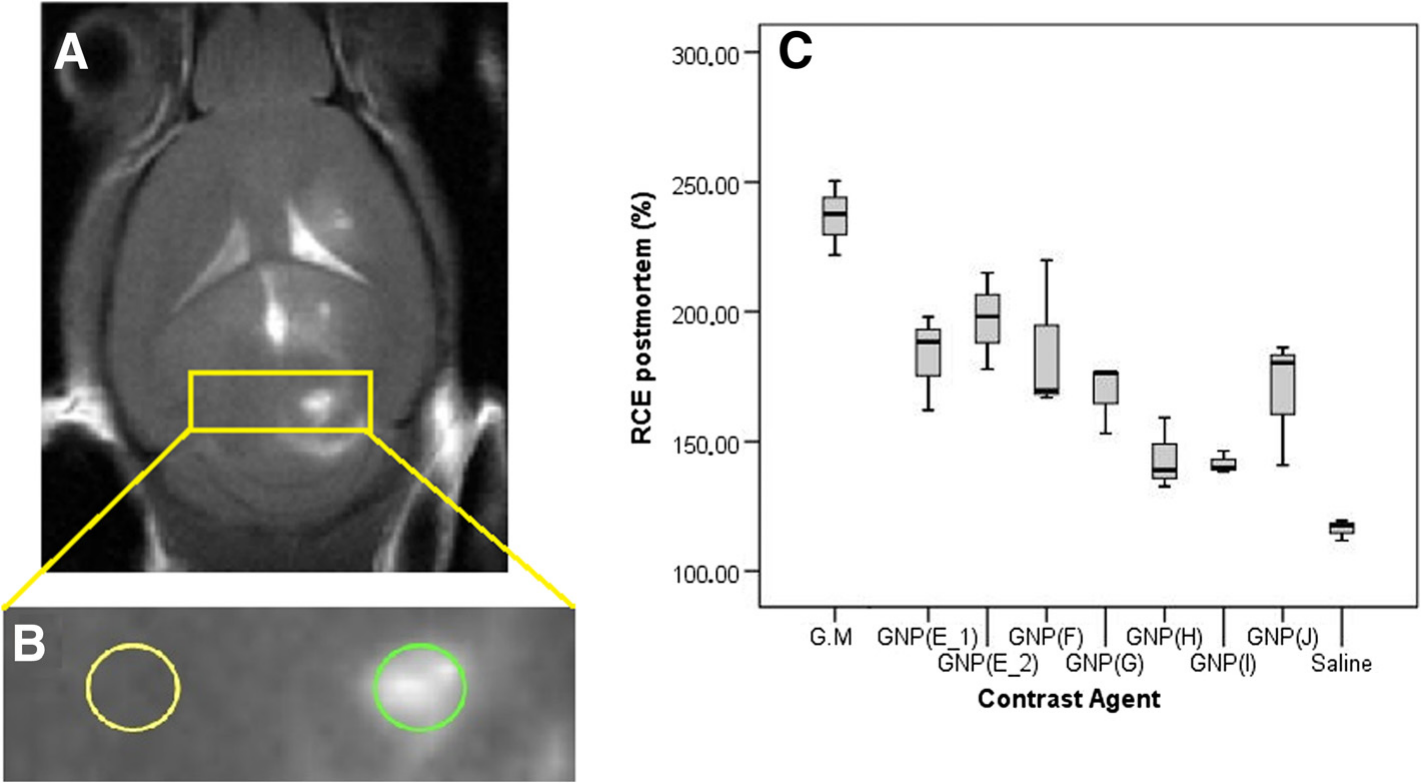
**Short name: *Ex vivo *MRI and example of Gd**-**GNPs evaluated; Boxplot of relative contrast enhancement.** Detailed legend: **A)** Representative *ex vivo* T_1_-weighted image and example of ROIs used (manually drawn as circles, green (ipsilateral) and yellow (contralateral) lines) for signal enhancement calculation for GNP (E_2). **B)** Enlarged central row (white discontinuous rectangle) **C)** Boxplot of relative contrast enhancement for saline solution, gadoterate meglumine (G.M.) and GNPs obtained from ROIs of all CAs studied. RCE was calculated using Equation 1. The limits of the box represent the quartiles 1 (Q1) and 3 (Q3) of the distribution, the central line corresponds to the median (quartile 2) and the whiskers represent the maximum and minimum value in each distribution. The data represented were included in the range [Q1-1.5 IQR - Q3 + 1.5 IQR], being IQR the interquartile range.

A representative T_1_-weighted image for Gd-GNP (E_2), as well as typical regions of interest (ROIs) selected for analysis, are shown in Figure 2A and 2B. The contrast enhancement obtained with this method clearly varies depending on the Gd-GNP investigated (Figure 2C). All Gd-GNPs investigated produced significantly lower contrast enhancement compared with gadoterate meglumine (236.7 ± 8.3% with respect to contralateral RCE, Table 1). The only exception was GNP (F) which was lower but not significantly. Among all GNPs, the GNP (E_2) presented the highest contrast enhancement (201.9 ± 9.3%). These results could be partially explained due to the differences in r_1_ and r_2_/r_1_ (Table 1) among the different CA. All GNPs but GNP (E_2) presented r_2_/r_1_ ranging from 5.00 to 11.06, clearly higher than the r_2_/r_1_ ratio measured for gadoterate meglumine (1.70). On the other hand, the r_2_/r_1_ ratio for GNP (E_2) was of 1.77. In addition, these results suggest that the sugar linker length could play a role in the results: GNPs with a C5 linker for sugars (GlcC5 (GNP E_1), GalC5 (GNP J)) tend to provide higher positive contrast enhancement (%RCE), when the chelating DO3A linker is C11 long. Still, GlcC2 (GNP F) also produced good results.

The *ex vivo* post-mortem MRI method was also successfully applied to iron-oxide nanoparticles. The results obtained have been summarized in the supplementary material (Section 2).

### *In vivo* MRI analysis

**Figure 3:**
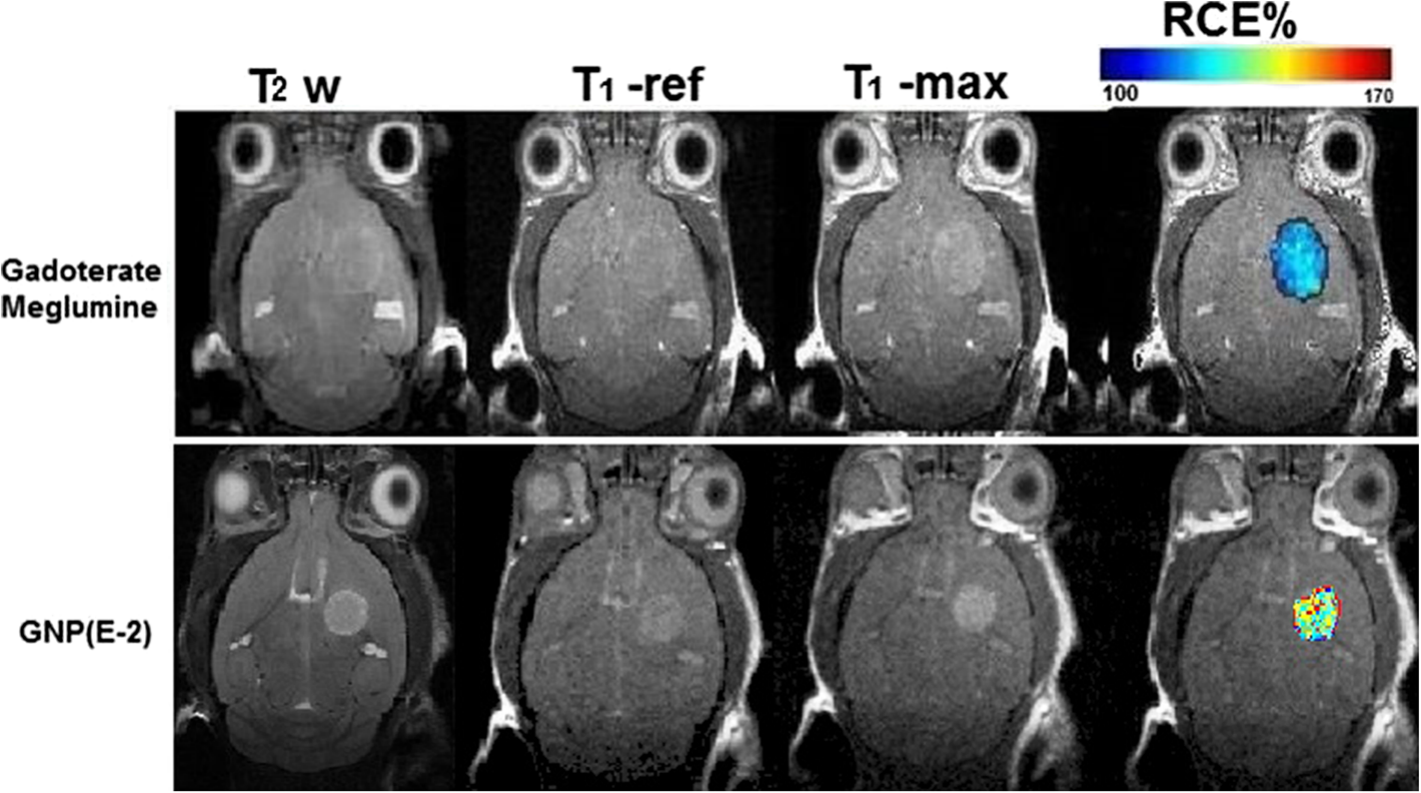
**Short name: Coronal T**_**2**_-**weighted images, DCE**-**MRI images and RCE maps of two mice bearing a GL261 glioma.** Detailed legend: From left to right, representative coronal T_2_-weighted images, DCE-MRI images (T_1_-weighted reference prior to contrast (T_1_-ref) and at the maximum contrast enhancement point (T_1_-max)), and RCE maps of two mice bearing a GL261 glioma. One animal was studied with gadoterate meglumine (top), and the other with GNP (E_2) (bottom). T_1_-ref images were acquired before injecting the contrast agent bolus while T_1_-max images correspond to the point of maximum contrast enhancement after gadoterate meglumine or GNP administration (see Figure 4). Colour coded bars on top of RCE provide intensity range shown in figures. Black pixels correspond to instances with values above or below the user-established %RCE postprocessing limits as described in methods.

**Figure 4:**
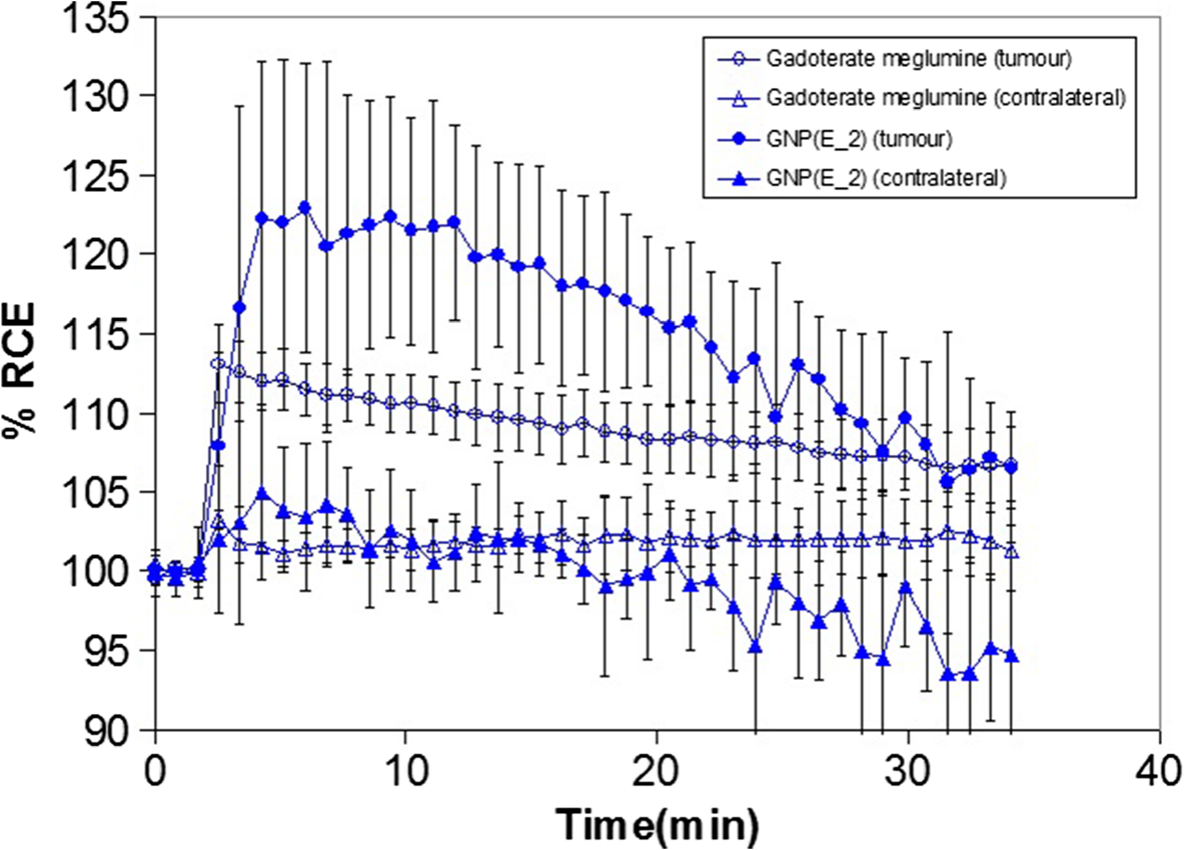
**Short name: RCE time**-**course curves obtained from the quantification of DCE**-**MRI images.** Detailed legend: RCE time-course curves obtained from the quantification of DCE-MRI images. Each curve displays the average contrast enhancement obtained for each group of mice. Values correspond to the results obtained from 3 animals/group and 3 slices/animal, i.e. n = 9 measurements/group. Gadoterate meglumine, open symbols; GNP (E_2), filled symbols. Tumor is coded by circles and contralateral non-tumoral areas by triangles. Bars show +/−SD.

T_2_-weighted images demonstrated the expected, predictable increase in tumor size and the overall morphology of a high grade glial tumor at 10 days post-inoculation of GL261 cells (Figure 3). The most consistent imaging finding in these tumors is the enhancement after contrast administration on post-contrast T_1_-weighted images. In this case, both contrast agents evaluated *in vivo* (gadoterate meglumine and GNP (E_2)) showed positive contrast enhancement on T_1_-weighted images (Figure 3, T_1_-ref vs.T_1_-max, and Figure 4).

**Figure 5:**
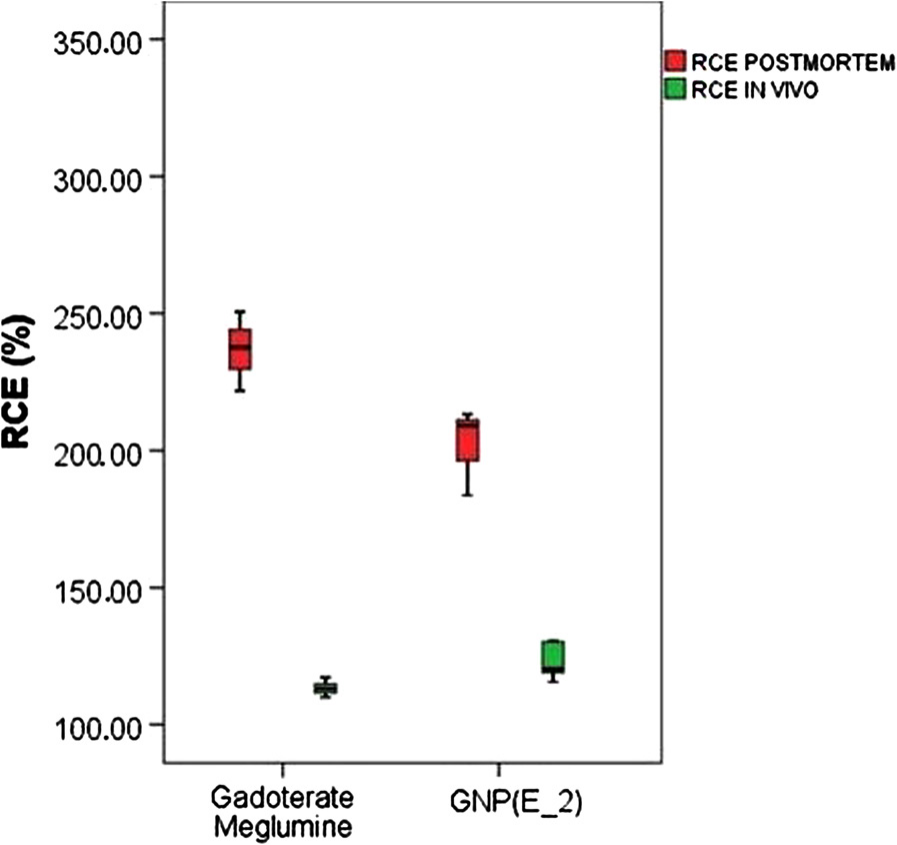
**Short name: Boxplot of %RCE obtained both *ex vivo *and *in vivo*at the T**_**1**_-**max time.** Detailed legend: Boxplot for comparison of %RCE obtained both *ex vivo* (red boxes) and *in vivo* (green boxes, at the T_1_-max time for each CA being studied) for gadoterate meglumine and GNP (E_2).

**Figure 6:**
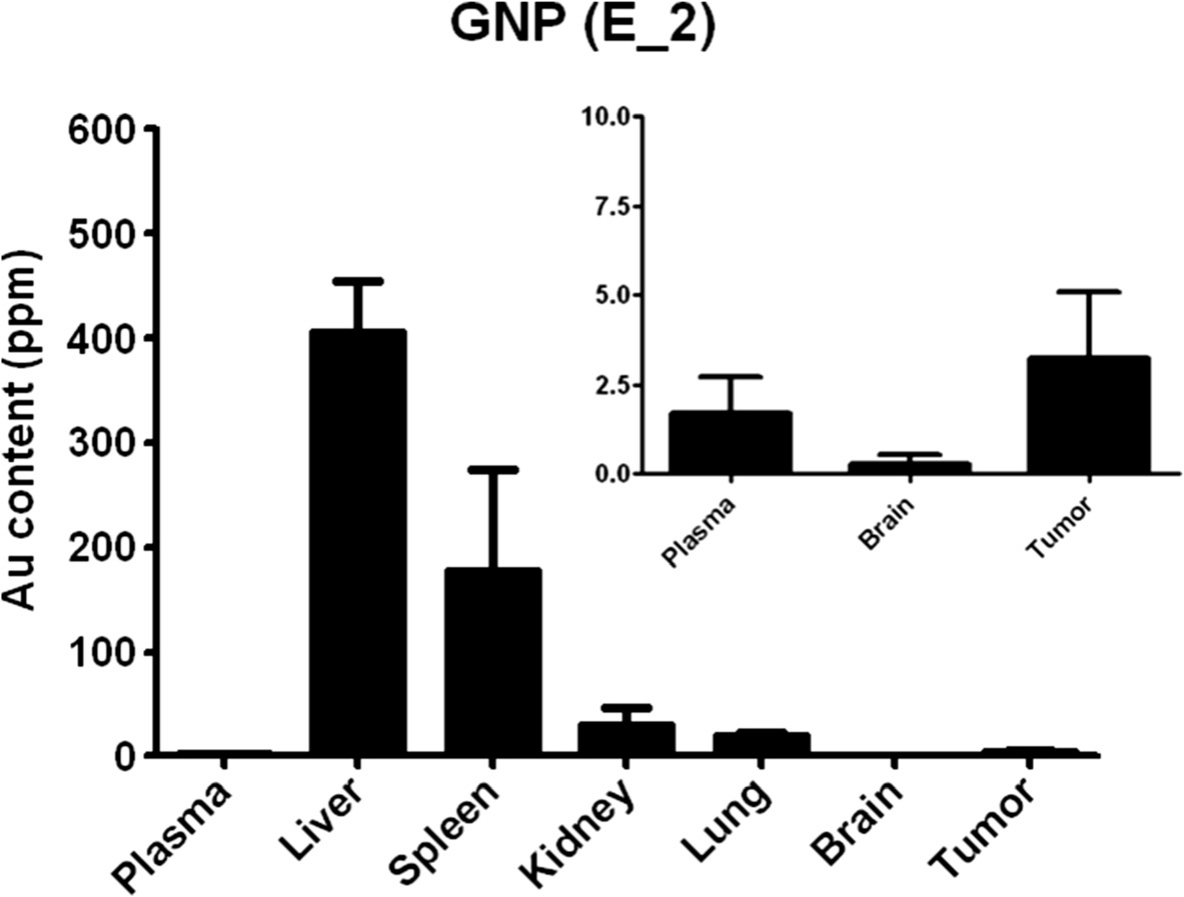
**Short name: Gold (Au) biodistribution as measured by ICP**-**MS 24 h post**-**administration of GNP (E**_**2).** Detailed legend: Gold (Au) biodistribution as measured by ICP-MS 24 h post-administration of GNP (E_2). Inset shows tumor Au accumulation at a different scale, compared to element concentrations in plasma and contralateral brain. The difference of Au accumulation between contralateral brain and tumor is nearly significant for GNP (E-2), *p* =0.056. Values are shown as mean parts per million (ppm) and SD (bar). Statistical comparisons correspond to unpaired t-tests.

Following the same trend observed in the *ex vivo* post-mortem studies, the GNP (E_2) showed a significantly (p < 0.05) worse performance in GL261 tumors (RCE 107.9 ± 5.9%) in comparison with gadoterate meglumine (113.1 ± 2.5%) when they were measured both at 2 min 56 sec post administration (corresponding to the time of maximum enhancement of gadoterate meglumine, Figure 4). The discrepancy between the *ex vivo* post-mortem studies (Table 1) and the information depicted in Figure 3 (RCE maps) is related to the time for maximum enhancement of each CA, which is significantly longer for GNP (E_2) (about 8 min) than for gadoterate meglumine (about 3 min) (p < 0.001), (Figure 4). If we take into account RCE at time of maximum enhancement for each CA (Figure 5), the GNP (E_2) outperforms gadoterate meglumine: RCE of 124.9 ± 8.3 and 113.1 ± 2.5%, respectively. *In vivo* imaging results were complemented by Induced Coupled Plasma-Mass Spectrometry (ICP-MS) measurements of gold content in tumor and normal brain (Figure 6). Indeed, Gd-GNP biodistribution assays after 24 h demonstrated that mouse brain tumors administered with GNP (E-2) accumulated almost ten times more CA than normal brain, and nearly twice the amount in circulating blood.

Renal and hepatic function parameters were also measured from the blood of animals administered with nanoparticles *in vivo*, including glucose, total proteins, albumin, creatinine, urea, total bilirrubine, aspartate aminotransferase (AST) and alanine aminotransferase (ALT)). Histopathological studies were also conducted, 24 h and 14 days post-administration of the CAs. No significant changes were found in any of the blood biochemical parameters analyzed in mice administered with GNP (E_1 or E_2) compared to control mice (only vehicle) as shown in Additional file 1: Table S1. Moreover, values for the three groups of mice remained within reference values [23,24]. Similarly, no alterations were found in histological sections of relevant organs such as liver, spleen and lungs (Additional file 1: Figure S2). Overall, these data suggest that the nanoparticles were non toxic to the animals in the time frame investigated although more detailed toxicological studies would be required in order to confirm (or refute) this assertion.

### Pharmacological parameters for CA tumor perfusion

**Figure 7:**
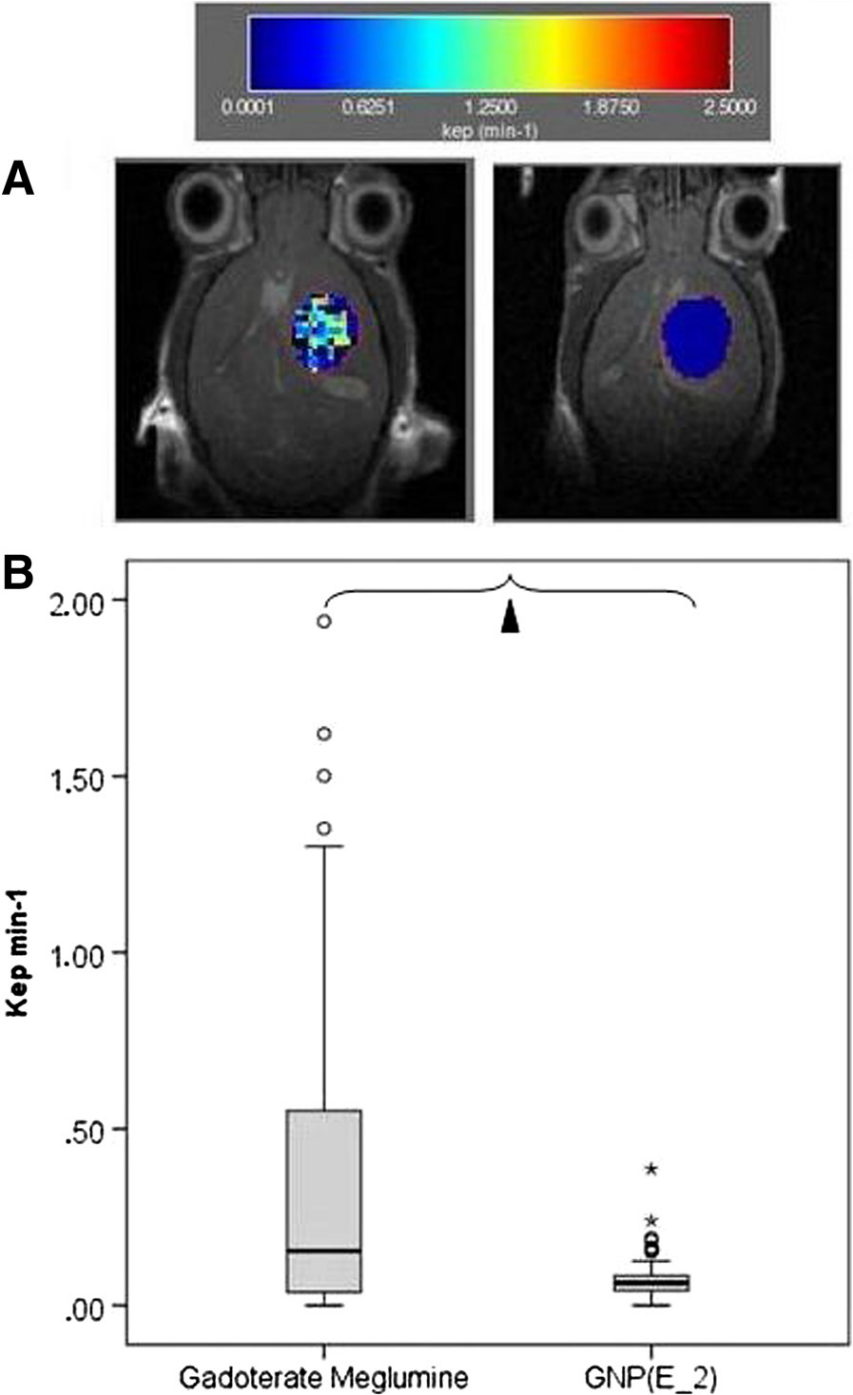
**Short name: Coronal *in vivo*T**_**1**_-**weighted MRI of mice bearing GL261 gliomas, with superimposed K**_**ep **_**colour**-**coded maps.** Detailed legend: **A)** Representative coronal *in vivo* T_1_-weighted images, with superimposed K_ep_ colour-coded maps of two mice bearing a GL261 glioma, administered with Gadoterate Meglumine (left), and GNP (E_2) (right). The black pixels correspond to instances with values above or below the user-established K_ep_ limits (see Methods). The color scale on top shows the range of values found for K_ep_ in each case, as well as their color coding. **B)** Boxplot for comparison of K_ep_ (min^−1^) obtained with all *in vivo* studied animals, using the same contrast agents shown in **A)**. Significant differences (p < 0.05) are marked with a triangle. The limits of the box represent the quartiles 1 (Q1) and 3 (Q3) of the distribution, the central line corresponds to the median (quartile 2) and the whiskers represent the maximum and minimum value in each distribution. The data represented were included in the range [Q1-1.5 IQR - Q3 + 1.5 IQR], being IQR the interquartile range. Outliers are represented outside the whiskers in two ways: as a circle if its value is in the range [Q1-1.5 IQR > value > Q1- 3.0 IQR or Q3 + 1.5 IQR < value < Q1- 3.0 IQR], or as an asterisk if values are above or below this threshold.

Table 2 and Figure 7 summarize % of RCE *ex vivo*, % of RCE *in vivo*, K_ep_ (exchange rate constant) and IAUC (initial area under the curve) for the gadoterate meglumine and the GNP (E_2), which had a full evaluation *in vitro*, *ex vivo* and *in vivo*.

**Table 2:** **RCE *ex vivo *, RCE *in vivo *, K**_**ep **_**and IAUC values obtained with *in vivo *** **studies**

**CA**	***% Ex vivo*** **RCE**	***% In vivo*RCE at T**_**1**_-**max**	***% In vivo*** **RCE at 3 min**	**K**_**ep**_**(min**^−**1**^**)**	**IAUC (A.U.)**
Gadoterate meglumine	236.7 ± 14.4	113.1 ± 2.5	113.1 ± 2.5	0.32 ± 0.36	2.8 ± 0.1
GNP (E_2)	201.9 ± 16.1	124.9 ± 8.3*	107.9 ± 2.0*	0.06 ± 0.03*	2.6 ± 0.2*

Detailed legend:

% RCE *in vivo* is shown both at T_1_-max of each CA and at 3 min after their administration.

* p < 0.05 in comparison with gadoterate meglumine.

## Discussion

Gadolinium complexes are widely used as positive CA in clinical MRI examinations. Besides the well known association of these agents with nephrogenic systemic fibrosis [25], they are non specific, i.e. able to detect anatomical and morphological changes but not a specific pathology. Developing contrast agents with specific targeting properties would have a significant clinical impact. One of the ways to approach this goal is to develop nano-platforms for attaching simultaneously a CA and targeting molecules forming nano-sized contrast agents [26-30].

The new positive CAs tested in this work are gold glyconanoparticles (GNPs) coated with multiple copies of sugars and Gd-complexes (Gd-GNPs) (Figure 1 and Table 1). They were prepared as T_1_-agents aiming to target mouse brain tumors [7,31] mimicking the multivalent presentation of carbohydrates at the cell surfaces [32,33]. These GNPs represent a novel multivalent platform for biological applications [29,34-37], soluble and stable under physiological conditions, and resistant to enzymatic degradation [38]. We selected simple carbohydrates to coat the Gd-based gold nanoparticles, as they are important nutrients for cells. Also, their uptake/binding is dependent on the expression of specific transporters (i.e. GLUTs) [39], and carbohydrate-binding proteins (lectins) which may confer targeting properties to the Gd-GNPs.

Different approaches have been tested to improve the assessment of new CAs, such as: studies with tumor-bearing animals, with no intermediate steps described between the CA evaluation *in vitro* and *in vivo* assays [40], computational simulation methods to predict the behaviour of a conditional CA [8], incubation of a CA with a gliosarcoma cell line in well plates to check for interaction and internalization using MRI [41], or incubation of a targeted CA with a cell line over-expressing the folate receptor in order to evaluate the efficiency of the targeting [42]. Those approaches based on *in vitro* relaxometry measurements incorporating the use of cell lines, are useful for high throughput CA analysis, but do not fully reproduce the *in vivo* environment [41]. In this respect, the interaction of the CA with extracellular macromolecules or proteins, may restrict the overall chelate mobility, and thus change its relaxivity [3]. Additionally, post mortem *ex vivo* approaches have been previously used to approximate the CA performance potential *in vivo*[11,12]. Those *ex vivo* studies [11,12] were conceptually similar to our herewith described method. Nonetheless, the tissues investigated by them (mouse leg [11] or subconjunctive eye tissue [12]), were not classical tissues for contrast enhancement experiments, as done in our case (brain/brain tumor). Authors in [11] did not proceed into comparing the performance of different CA or validated the possible relevance in an *in vivo* model. Furthermore, authors in [12] did compare *ex vivo* post-mortem data with *in vivo* data, but the emphasis was placed in CA clearance rate changes between low and high molecular weight CA rather than in the development and evaluation of a method to gauge contrast potential among different agents. Generally speaking, several effects related to the *in vivo* environment cannot be easily evaluated *in vitro*. Accordingly, we believe that this biological environment should be mimicked as closely as possible in order to achieve an optimal prediction of the CA potential. In this sense, our method should perform better than previously described strategies [8,11,12,38-40]. The results obtained in this study, which will be discussed next, suggest the importance of taking into account not only these CA *in vivo* environment effects because other factors, such as Gd loading and macromolecular behaviour of the CA, could also play a role and should be considered as relevant.

### Effect of nanoparticle coating in positive CA results

We have previously observed that small modifications in the chemical structure of ligands coating the Gd-GNPs lead to changes in its contrast enhancement effects [7]. This could be due to the Gd hydration number or the overall CA correlation time which, in turn, could affect their relaxivity.

When considering the *in vitro* relaxivity measurements described in this work, differences (see Table 1) were observed among the GlcCX-DO3AC11 series (GNPs (E_1), (E_2) and F-J, X being a number between 2 and 9). These differences could be influenced by the length of the saccharide’s linker, which could modulate the access of water to the Gd chelated ion. As an example, GlcC3-DO3AC11 (GNP I) has the lowest r_1_ value at 1.4 T (for glucose GNPs) and tends to be in the low range values region at 7.0 T. On the other hand, there was a tendency to obtain better r_1_ values at 1.4 T with C5 linkers (e.g. GNPs (E_2) and J) and the same tendency is observed at 7.0 T, despite a slightly different behaviour is seen in GNP (E_1).

The *ex vivo* post mortem studies showed discrepant results compared to *in vitro* r_1_ values: the GlcC5 linker did not produce a high RCE, as expected from the *in vitro* r_1_ results. In this sense, the *in vitro* high relaxivity values measured for the GNPs (E_2) and (J) did not turn into high contrast enhancement *ex vivo*. It is also feasible that the Gd loading (between 3.2 and 7% Gd in weight of nanoparticle, see Table 1 for details) of these GNPs, could have an influence in the RCE obtained in each case, even if correcting the preparation of the CAs solutions to achieve the same final concentration of Gd for *ex vivo* post mortem studies. In this respect, the percentage Gd content per nanoparticle significantly correlates with r_1_, but not r_2_ (Additional file 1: Figure S1). Thus the beneficial effect of high payload per nanoparticle has been demonstrated for positive contrast targeted nanoparticles in *ex vivo* studies with cultured cells [43]. This fact could also explain the differences between GNPs (E_1) and (E_2), which have the same structural formula but different percentage of Gd (per mass of GNP), and different r_1_ values *in vitro as* well as different RCE *ex vivo*. Moreover, the nature of the carbohydrate may play a role in the *ex vivo* post-mortem RCE. This could be observed for GNP (J), coated with galactose, where the high r_1_ values at 1.4 T and 7.0 T did not turn into high RCE *ex vivo*. It is also worth pointing out the possible r_2_ contribution which could counteract the r_1_ effect [44], and, therefore, the overall RCE (e.g. GNP (E_1), r_2_/r_1_ = 7.48 and %RCE = 182.8; GNP (E_2), r_2_/r_1_ = 1.77 and %RCE = 201.9, see Table 1 for further details).

Additionally, the length of the alkane linkers for glucose may modulate the interactions with the cellular receptors, and, accordingly the targeting of the investigated nanoparticles. It is well known that GLUT-1 transporters for glucose are overexpressed in tumor cells [45] and its structure has been extensively investigated. In this respect, an “optimal” length of the saccharyde linker in the CA glucose series may be required to properly interact with the GLUT-1 transporters, which would translate into maximum restriction of the Gd^3+^ chelate mobility and low correlation times. The length of the alkane linker may modulate these interactions. In this sense, the “C3-linker” (GNP I) produced one of the lowest *ex vivo* RCE values measured in the GlcCX-DOTAC11 series. We also found significant differences compared to C5 and C9 linkers (GNP (E_1) and GNP (G)). This would suggest that a glucose linker arm too short could compromise proper interaction of the GNP with GLUT transporters, although additional studies will be needed to fully evaluate this possibility. Furthermore, results in [46] provide some support to the “targeting” hypothesis by showing that glucose-coated niosomes produced contrast-enhancement, whereas niosomes without glucose produced no enhancement, reinforcing the possibility that this interaction nanoparticle-transporter is also present in our case. Besides to possibly modulating *ex vivo*/*in vivo* CA relaxivity values, the use of glucose could also help targeting the CA-containing nanoparticles to tumor cells by means of their glucose transporters.

Preliminary results comparing the *in vivo* effects of a similar GNP (with the same structural formula as GNP E_1 and E_2) in the same tumor model did not show significant differences in the maximum RCE changes observed during different glycemic states, euglycemia and hyperglicemia (26.9 ± 1.8 mM in blood) (results not shown). This suggests that the possible competition between the glucose GNPs and free glucose for the interaction with GLUT transporters does not seem to affect their performance as T_1_ contrast agents. Nonetheless, further assays on the *in vivo* competition between glucose GNPs with and without chelated Gd should help to clarify the point about the contribution of the glucose GNP binding to GLUTs in the measured RCE.

The *in vitro* r_1_ relaxivity value for GNP (E_2) at 7 T was the best among the Gd-GNPs (Table 1), but the *ex vivo* analysis did suggest slightly, but significant, worse performance than gadoterate meglumine. The *ex vivo* and *in vivo* RCE measurements both predicted worse performance of GNP (E_2) compared to gadoterate meglumine when RCE was measured at the same time point (3 min post CA injection, time of maximum contrast enhancement for gadoterate meglumine) (Table 2). However, when RCE was measured at the time of maximum enhancement for each CA in the DCE-MRI experiments, the GNP (E_2) performed significantly better than gadoterate meglumine (see Table 2 and Figure 5). Significant differences were found in the maximum time of contrast enhancement (“T_1_-max”) between the two analyzed CA, and this points to the fact that the behaviour of GNP (E_2) is the expected one for a macromolecular-like contrast agent [12]. This may be due to the well known “enhanced retention” (EPR) effect of nanoparticulated CAs in tumors [47]. The described diameter of the GNPs is between 1.8-4.5 nm [7], comparable to agents used as macromolecular contrast agents (4–6 nm, [12,48]), and therefore reach the time to maximum enhancement more slowly than free gadolinium chelate (gadoterate meglumine) (Figure 4). This result agrees with previous results obtained using niosomes by authors in [46] although with a different tumor type. Finally, the gold biodistribution found in our study (e.g. maximal accumulation found in liver and spleen, Figure 6) is in good agreement with literature for gold nanoparticles [49,50].

The pharmacological parameters measured for gadoterate meglumine and GNP (E_2) (e.g. K_ep_, see Figure 7 and Table 2) were significantly different. Authors in [12] have previously shown that CAs with different molecular sizes also have different clearance rates (lower K_ep_ values), which becomes slower with higher molecular weight CAs. Authors in [48] have measured a closely related parameter, the “transfer coefficient” (K_PS_) (transfer rate of the contrast agent from the blood to the interstitial space, highly dependent on the permeability), and found an inverse relationship between the molecular weight of the CA and the transfer coefficient. Then, GNP (E_2) behaves as a macromolecular contrast agent, according to their average K_ep_ values.

Finally, in addition to the more accurate simulation of the *in vivo* conditions, the *ex vivo* post mortem method described here consumes a very small amount of CA compared to an *in vivo* study: 800 times less in the case of positive contrast agents (5 nmol/animal vs. 4,000 nmol/animal) and it is applicable both to positive and negative (Supplementary Material and [51]) CA. Furthermore, as it is carried out with sacrificed animals, no preliminary toxicological assays are required. It is worth pointing out that, although this method is better at simulating the *in vivo* conditions, the use of animals is costly and it would require certain facilities and compliance of legal/ethical aspects, depending on the country were researchers are based. The development of new and improved *in vitro* strategies, able to evaluate cells exposing groups or proteins able to be targeted, could also offer an intermediate and useful step in this field.

## Conclusions

In summary, we have developed a low-consumption *ex vivo* method for testing the potential *in vivo* performance of new contrast agents. This should allow us to choose from a series of new candidate CAs the one/s more likely to perform better *in vivo*. This method requires small amounts of CA (e.g. 5 nmol/animal) and should allow a more rationally informed selection, avoiding unnecessary *in vivo* and toxicology tests for the *ex vivo* poorly performing substances. This reduces animal needs, material and overall costs. Nevertheless, caution should be taken when evaluating CAs with different kinetic behaviour (e.g. small vs. high molecular weight CAs). In this case, the *ex vivo* method could produce an underestimation of the actual contrast enhancement potential. Further studies may be needed in order to fully optimize the currently described protocol.

## Methods

### Synthesis of contrast agents

#### Gd-complexes containing glyconanoparticles (Gd-GNP)

The positive contrast agents (Gd-GNPs) evaluated in this work were 1.8-4.5 nm sized gold nanoparticles coated with multiple copies of sugar conjugates and 1, 4, 7, 10-tetraazacyclododecane-1, 4, 7-triacetic acid (DO3A) Gd-complexes. The basic structures and gadolinium content of each Gd-GNP type are schematized in Figure 1 and listed in Table 1. The Gd-GNPs were prepared by ligand place exchange reaction (LPE) starting from glyconanoparticles (GNPs) coated only with sugars as described in [13,31] (Gd-GNPs E_1, E_2 and F-J). The sugars (glucose, galactose) are conjugated to an aliphatic chain of variable number of carbon atoms (C2, C3, C5, C7, C9, C11), while the DO3A derivatives contain a C11 aliphatic chain (Table 1). The obtained Gd-GNPs were characterized by TEM, UV–vis, IR, ^17^O NMR and ICP-AES as described in [7] and [31]. The average diameter of the gold core was 1.5-2.5 nm and the number of sugars and Gd chelates on the surface ranged between 70–130 molecules for sugars and 14–34 for Gd chelates. The amount of Gd (III) was between 3.2% and 7.0%, determined by ICP-AES (induced coupling plasma-absorption spectroscopy).

### *In vitro* relaxivity studies

#### Studies at 1.4 T

The longitudinal T_1_ relaxation times of Gd-GNPs were determined at 10, 5, 2.5, 1.25, 0.625 mg mL^−1^ concentrations of Gd-GNPs in water (Gd concentration ranged from 0 to 3 mM) at 60 MHz (1.41 T) and 37°C in a Bruker Minispec MQ-60 NMR spectrometer (Bruker Optik, Ettlingen, Germany) at CICbiomaGUNE, San Sebastián, Spain. The values of T_1_ were determined using the inversion-recovery method. The calculation of the T_1_ was carried out using monoexponential fitting. Three independent measurements of T_1_ were performed in every sample for statistical analysis purposes.

Relaxivities r_1_ and r_2_ were obtained from the slopes of the curves 1/T_1_ and 1/T_2_ vs the concentration of Gd expressed in mM.

#### Studies at 7.0 T

Four different solutions ranging from 0.125 to 1 mM in gadolinium of Gd-GNPs and gadoterate meglumine in saline solution (NaCl 0.9%, B.Braun) were used. A phantom using capillary tubes (1.5 mm internal diameter) for GNP solutions, immobilized in plasticine was used for MR scanning.

All 7 T studies (*in vitro* relaxivity, *ex vivo* post mortem and *in vivo*) were carried out at the joint NMR facility of the Universitat Autònoma de Barcelona and CIBER-BBN (Cerdanyola del Vallès, Spain), using a 7 T horizontal magnet (BioSpec 70/30; Bruker BioSpin, Ettlingen, Germany) equipped with actively shielded gradients (B-GA12 gradient coil inserted into a B-GA20S gradient system). A quadrature 72 mm inner diameter volume coil was used for *in vitro* studies whereas a quadrature receive surface coil, actively decoupled from a linear volume resonator with 72 mm inner diameter was used for *in vivo* and *ex vivo* studies. The *in vivo* studies were performed at 37°C, whereas the *in vitro* relaxivity and *ex vivo* post mortem studies were performed at 23°C. A recirculating water-system, incorporated to the animal bed, was used to control the temperature, measured by a probe and was constantly monitored (SA Instruments, Inc., New York, USA).

All studies started with acquiring fast T_2_-weighted images in three orthogonal planes using a Rapid Acquisition by Relaxation Enhancement (RARE) sequence; Rare Factor, 8; field of view (FOV), 19.2×19.2 mm; matrix size (MTX), 128×128 (150×150 μm/pixel); number of slices (NS), 14 (coronal), 7 (sagittal) and 5 (axial); slice thickness (ST), 1 mm; interslice-thickness (IST), 1.1 mm; TR/TE, 2000/36 ms; number of averages (NA), 1; total acquisition time (TAT), 24 sec.

After these initial sequences, relaxivity studies (T_1_ and T_2_ maps) for Gd-GNPs were carried with the following parameters:

For T_1_ maps acquisition, a RARE-VTR sequence was chosen with RARE factor = 4; FOV, 7.0×3.5 cm; MTX, 512×256 (137×137 μm/pixel); NS, 1; ST, 1 mm; TE_eff_, 30 ms; TRs were according to the following list: 75, 150, 300, 600, 1200, 2400 and 4800 ms; NA, 2; TAT, 20 min.

For T_2_ maps acquisition, a Multi-Slice-Multi-Echo (MSME) sequence was used; FOV, 7.0×3.5 cm; MTX, 512×256 (137×137 μm/pixel); NS, 1; ST, 1 mm; TR, 4800 ms; TEs were according to the following list: 20, 40, 60, 80, 100, 120, 140, 160, 180, 200, 220, 240, 260, 280, 300, 320, 340, 360, 380 and 400 ms; NA, 4; TAT, 1 h 16 min.

### Animal models

The animal model used for these studies was C57BL/6 healthy female control mice of 20–25 g weight. All C57BL/6 animals were obtained from Charles River Labs (France) and housed at the Animal Facility of the *Universitat Autònoma of Barcelona*.

For the *ex vivo* post mortem studies n = 8 (one for each CA) were used for the CA characterization procedures. For the *in vivo* study, n = 6 mice harbouring a GL261 glioblastoma were used. For the orthotopic model generation, the GL261 mouse glioma cell line was obtained from the Tumour Bank Repository at the National Cancer Institute, Frederick, MD, USA. Cells were grown in RPMI-1640 culture medium supplemented with 2.0 g/L sodium bicarbonate, 0.285 g/L L-glutamine, 10% foetal bovine serum and 1% penicillin-streptomycin solution. Culture medium and chemicals were purchased from Sigma-Aldrich (St. Louis, MO, USA) unless otherwise indicated. Culture plastic was obtained from Nunc (Roskilde, Denmark).

Tumors were induced in the six mice by intracranial stereotactic injection of GL261 cells into the caudate nucleus, essentially as described in [52]. MRI studies were performed approximately 2 weeks after cell inoculation.

All studies were carried out according to protocols approved by the local/institutional ethics committee, according to the regional and state legislation (CEEAH 1178 and 1176).

### *Ex vivo* and *in vivo* MRI studies

These studies were carried out at the joint NMR facility of the Universitat Autònoma de Barcelona and CIBER-BBN (Cerdanyola del Vallès, Spain), using a 7 T horizontal magnet.

All studies started with high resolution T_2_-weighted images which were acquired from three horizontal sections of the brain in order to have a good morphological characterization of the investigated tissue. The sequence used for this purpose was RARE with the following parameters: RARE factor = 8, FOV, 17.6×17.6 mm; MTX, 256×256 (69×69 μm/pixel); ST, 1 mm; NS, 3; TR/TE, 3000/36 ms; NA, 4; TAT, 4 min 48 sec.

### *Ex vivo* post mortem studies

The CAs to be used for post mortem *ex vivo* evaluation were dissolved in saline solution (0.9% NaCl, B.Braun) taking into account the estimation of Gd content previously determined (see Table 1), in order to achieve solutions of comparable concentrations of the desired metal. The amount finally used for each animal was 5 nmol of Gd dissolved in 4 μl of saline solution. A commercial solution of Gd CA (Gadoterate Meglumine, DOTAREM®, Guerbet, Roissy, France) was used as a standard to compare the enhancement obtained by MRI with the GNPs.

A T_1_-weighted image acquisition was performed using the same coronal sections described previously in “*in vitro* 7 T studies”. For this, a MSME sequence was used with: FOV 17.6×17.6 mm; MTX, 128×128 matrix (138×138 μm/pixel); TR/TE, 350/8.5 ms; NA, 1; number of repetitions (NR), 15; TAT, 11 min. Animals were anesthetized and handled as described for “*in vivo* studies”. After that, animals were sacrificed with an overdose of intraperitoneal sodium pentobarbital (200 mg/kg) (Vetoquinol, Madrid, Spain), and then immobilized on a stereotactic holder (Kopf Instruments, Tujunga, CA, USA). The contrast administration was carried out as described for tumor generation [52], except that the cell suspension was replaced with the contrast agent solution to be evaluated, and 3 injection points at each side were administered, as follows: 1.0 mm holes were made lateral (right) to the midline, 2.32/2.32/2.00 mm, and with the following “Y” coordinates along the midline: 0.10/1.5/4.5 mm. The whole process of CA injection *ex vivo* took 15 minutes. After this, the T_1_ weighted image acquisition was repeated as above.

### *In vivo* studies

Anesthesia was performed with isoflurane (B.Braun, Melsungen, Germany) at 0.5-1.5% in O_2,_ maintaining the respiratory frequency between 40–60 breaths/min. Body temperature was maintained between 36.5-37.5°C with a recirculating water system incorporated in the animal bed, and measured with a rectal probe. Respiration rate and temperature were constantly monitored (SA Instruments, Inc., New York, USA). Before immobilization in the animal holder, each mouse was cannulated in the tail vein using a home-built multi-delivery polyethylene tubing system. In this case, a 30G 2-way catheter was connected, through polyethylene tubing, to 2 independent 1 mL syringes (Becton-Dickinson S.A., Madrid, Spain) loaded with heparinized-saline (40 U/ml) (0.9% NaCl, B.Braun and heparin, Mayne Pharma España, Madrid, Spain) and one contrast agent (9.7 mM in Gd). The contrast agent was injected into the mice as a bolus (72–84 μL, about 0.04 mmol/kg) during Dynamic Contrast Enhanced (DCE)-T_1_ MRI studies. Three glioma-bearing mice were injected with gadoterate meglumine and another three were injected with GNP (E_2) (see Results).

A DCE T_1_ study was then performed using three coronal sections. For this, a MSME sequence was used with: MTX, 128×128 matrix (138×138 μm/pixel); TR/TE, 200/8.5 ms; ST, 1 mm; NA, 2; NR, 41; TAT, 35 min. The contrast bolus was administered after the third repetition of the complete T_1_-weighted sequence (about 2.5 min after the start of the image acquisition protocol).

### Biodistribution of Gd-GNP by ICP-MS

At established time-points (24 h, 48 h, see further details in the Additional file 1: Table S1) after GNP or vehicle administration, animals (n = 3 for each time point) were euthanized by cervical dislocation and required tissues (liver, kidney, spleen, brain and tumor among others) as well as urine and blood samples were collected and stored at -80°C. Samples were analyzed by Induced Coupled Plasma-Mass Spectrometry (ICP-MS) at the *Unidad de Análisis Elemental* of the Scientific Technical Facilities of the University of Barcelona.

### Histopathological analysis

At established time-points (24 h, 48 h and 14 days) after GNP or vehicle administration administration, animals (n = 3-6 for each time point) were sacrificed by cervical dislocation. The required tissues were dissected, fixed and stained with Haematoxilin-Eosin.

### Processing and post-processing of MR data

*Ex vivo* post mortem studies: all processing and post-processing of T_1_ -weighted images were carried out with Bruker software Paravision (version 4.0) and ImageJ 1.44p (National Institutes of Health, USA^a^). Three regions of interest (ROIs), corresponding to the coordinates described in the “*ex vivo* post-mortem studies” section were manually defined after visual inspection both in the area of maximum enhancement and equivalent area of contralateral parenchyma. The relative contrast enhancement (RCE) - injection site ROI vs. contralateral parenchyma - obtained in each case was used for calculations (see Equation 1). Only the slice with better defined contrast-enhanced region was measured.

(1) $$\mathrm{RCE}{\left(\%\right)}_{\mathrm{ex}\_\mathrm{vivo}}=\left(\frac{S\left(i\right)}{S\left(c\right)}\right)\times 100$$

Where S (i) is the absolute signal intensity of the “ipsilateral” region with respect to the contrast administration and that visually shows contrast enhancement, and S (c) is the absolute signal intensity of the equivalent contralateral region, which serves as a control and that is defined as “100%”.

*In vivo* studies: DCE-MRI data were processed using a platform for pharmacokinetic analysis recently developed within the intramural CIBER-BBN project PROGLIO/PROGLIO2 using IDL (ITT Visual Information Solutions, Boulder, CO, USA) available for download at http://www.die.upm.es/im/archives/DCEurLAB/ from the Biomedical Images Technologies research group of the *Universidad Politécnica de Madrid* (BIT-UPM) [53], and also with additional home written scripts in IDL as described in [54]. RCE *in vivo* was calculated for each pixel as the ratio between the maximum signal enhancement and the average intensity before bolus injection unless otherwise indicated, and the three slices acquired were used for this calculation. In this way, time-course curves that quantified the average contrast enhancement inside the tumors and in surrounding non tumor-bearing areas of the brain parenchyma (defined from ROIs) were generated. Moreover, color-coded maps that translated the maximum contrast enhancement at each pixel of the FOV at the time of maximum enhancement were also generated (RCE). Maps with the initial area under the curve (IAUC) were generated by integration of the dynamic curves during the first 150 seconds after contrast agent administration, as in [55,56] but without normalization to a reference IAUC value – a constant arterial input function was assumed. K_ep_ parameters (Eq. 2) were calculated according to the “Hoffmann-Brix” model [57]:

(2) $$\frac{S\left(t\right)}{S\left(0\right)}=1+Ak_{\mathrm{ep}}\frac{\left(e^{-k_{\mathrm{ep}}t}-e^{-k_{\mathrm{el}}t}\right)}{k_{\mathrm{ep}}-k_{\mathrm{el}}}$$

being K_el_ the elimination rate of the contrast agent by the plasma. Thus, the initial slope of the kinetic curve, detected at short times after injection, is proportional to A.K_ep_, being A the initial upslope of the enhancement curve.

For each animal and slice, ROIs were manually drawn around the tumor. The resolution of all datasets was reduced from 128×128 to 64×64 pixels and RCE, IAUC, and K_ep_ maps were generated within those ROIs. The maps were coloured according to a scale comprising the maximum and minimum values manually defined. Pixels with values above or below these limits were coloured black. The whole adjustment of each pixel (including fitting deviation and mean errors) could be exported in an excel format. This helped improving the accuracy of the “Hoffmann-Brix” model, allowing us to detect inadequate fittings which were filtered-out by defining thresholds for standard deviation (< 0.5) and mean error (< 1.5), above which pixels were discarded from the analysis. Statistical analysis within the tumor regions on each slice were carried out with SPSS *19.0* (SPSS Inc., Chicago, USA). A total of 836 and 699 pixels were analyzed from animals studied with gadoterate meglumine and GNP (E_2) injection, respectively.

### Statistical analysis

*Ex vivo* post mortem studies: the overall signal change measured in T_1_ weighted MRI ROIs for each GNP was compared with gadoterate meglumine [7,58] and saline solution.

*In vivo* studies: measurements from the same animal but corresponding to different slices were considered as independent measurements/cases (total n = 9 for each CA). For *in vivo* studies, the average contrast enhancement in the ROI inside the tumors was also compared among animals injected with different contrast agents.

For both studies, normality was first inspected in each group by the Kolmogorov-Smirnov test and variance homogeneity with the Levene test. A two-tailed Student’s t-test for independent measurements was used for statistical analysis when data followed a normal Gaussian distribution. If data had a non-normal distribution, Mann–Whitney U test was used for statistical analysis. The significance level for all tests was p < 0.05.

## Endnotes

^a^http://rsb.info.nih.gov/ij/.

## Abbreviations

A.Kep: Amplitude times exchange rate constant K_ep_; BBB: Blood brain barrier; CA: Contrast agent; DCE: Dynamic contrast enhancement; DMSA: Dimercaptosuccinic acid; FOV: Field of view; GBM: Glioblastoma; Gd-GNP: Gadolinium-based glyconanoparticle; GM: Gadoterate meglumine; IAUC: Initial area under the curve; ICP-AES: Induced coupling plasma-atomic emission spectroscopy; IDL: Interface description language; IR: Infrared spectroscopy; IST: Inter-slice distance; Kel: Elimination rate constant by the plasma; Kep: Efflux rate constant from extravascular extracellular space to plasma; MRI: Magnetic resonance imaging; MSME: Multi-slice-multi-echo; MTX: Matrix size; NA: Number of averages; NEX: Number of excitations; NR: Number of repetitions; NS: Number of slices; PBS: Phosphate-buffered saline solution; RARE: Rapid acquisition by relaxation enhancement; RCE: Relative contrast enhancement; ROI: Region of interest; SI: Signal intensity; SPION: Superparamagnetic iron oxide nanoparticles; ST: Slice thickness; TAT: Total acquisition time; TE: Echo time; TEeff: effective echo time; TEG: Triethylene glycol; TEM: Transmission electron microscopy; TR: Recycling time; UV–vis: Ultraviolet–visible spectroscopy.

## Competing interests

The authors declare that they have no competing interests.

## Authors’ contributions

MA, RVS and TDG performed the acquisition of *ex vivo* and *in vivo* MRI data, as well as relaxivity data at 7 T. SLP set up the 7 T MRI/MRS sequences. AI and MM carried out the glyconanoparticle synthesis, evaluation and relaxivity studies at 1.4 T. SP was responsible for design and supervision of glyconanoparticle synthesis and characterization. IA and SSJr performed the biodistribution studies. NMS and OBM participated in SPION synthesis and characterization, as well as relaxivity studies at 1.4 T. JS was responsible for design and supervision of SPION synthesis and characterization. CA was responsible for the design of the experimental part of the study and also in the analysis and interpretation of critical data. APC participated in the supervision of preclinical studies and acquisition of MRI *in vivo* data, being also responsible for the first draft of the manuscript. All authors participated in the revision and approval of the manuscript final version.

## Supplementary Material

Additional file 1 **1. Biodistribution and toxicological studies carried out with GNP E_1 and E_2.** 2. Application of the *ex vivo* postmortem method to iron oxide nanoparticles. Click here for file
