# Supplementary material for: A new ex vivo method to evaluate the performance of candidate MRI contrast agents: a proof-of-concept study
Source: J Nanobiotechnology. 2014 Apr 5;12:12. doi: 10.1186/1477-3155-12-12 (PMC4021710; doi:10.1186/1477-3155-12-12)
Supplement: Additional file 1 — 1. Biodistribution and toxicological studies carried out with GNP E_1 and E_2. 2. Application of the ex vivo postmortem method to iron oxide nanoparticles. [file 1477-3155-12-12-S1.pdf]

A new *ex vivo* method to evaluate the performance of candidate MRI contrast agents: a proof-of-concept study

Candiota AP, Acosta M, Simões RV, Delgado-Goñi T, Lope-Piedrafita S, Irure A, Marradi M, Bomati-Miguel O, Miguel-Sancho N, Abasolo I, Schwartz S Jr, Santamaria J, Penadés S, Arús C

SUPPLEMENTARY MATERIAL

1. Biodistribution and toxicological studies carried out with GNP E_1 and E_2

Methods

Contrast Agents

The positive contrast agents (Gd-GNPs) evaluated in this work were 1.8-4.5 nm sized gold nanoparticles coated with multiple copies of sugar conjugates and 1, 4, 7, 10-tetraazacyclododecane-1, 4, 7-triacetic acid (DO3A) Gd-complexes (see reference [1] and table 1 of the main article for further details). See reference list for the supplementary material at the end of the document.

The Gd-GNPs were prepared by ligand place exchange reaction (LPE) starting from glyconanoparticles coated only with sugars as already described in the main text of the article (e.g. GNP (E_2)).

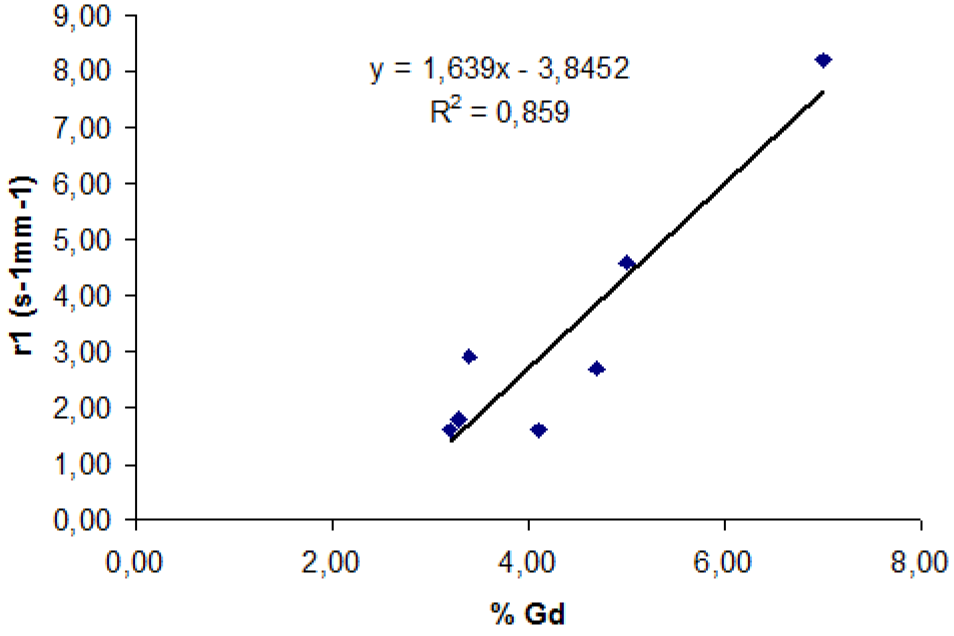

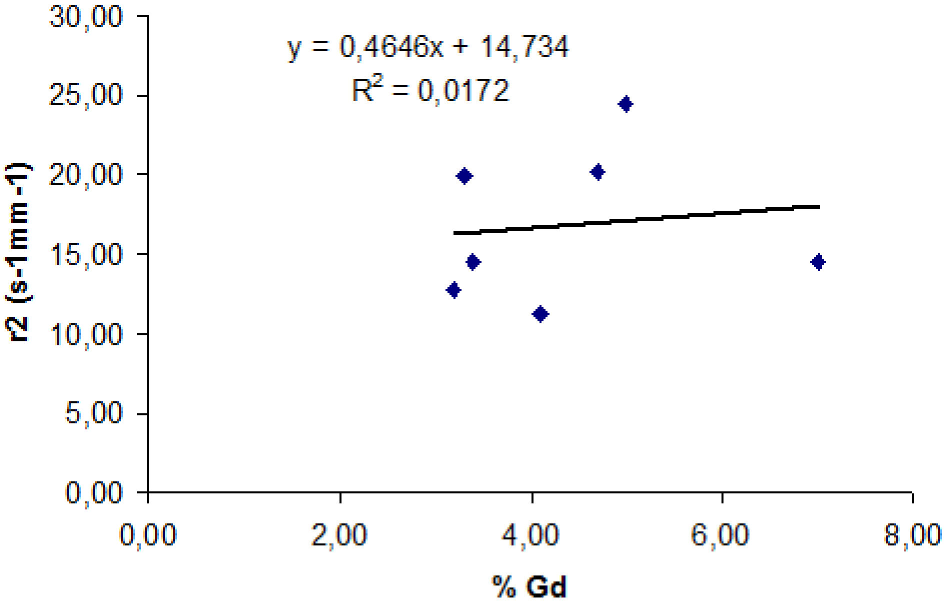

Supplementary Figure 1. Correlation of % of Gd in the Gd-GNPs (see Table 1) and A) r_1 relaxivity ($s^{-1}.mM^{-1}$) and B) r_2 relaxivity ($s^{-1}.mM^{-1}$), from Table 1, both measured at 7T.

Biodistribution of Gd-GNP by ICP-MS

At established time-points after i.v. injection GNP or vehicle (Phosphate Buffered Saline, PBS) administration animals (n=3 per group, as mentioned in methods: “MRI studies – *in vivo* studies”) were sacrificed by cervical dislocation. Required tissues (liver, kidney, spleen, brain and tumor among others) as well as urine and blood samples were collected and stored at -80ºC. Samples were analyzed by Induced Coupled Plasma-Mass Spectrometry (ICP-MS) at the *Unidad de Análisis Elemental* of the Scientific Technical Facilities of the University of Barcelona.

Supplementary Table 1: Blood biochemical parameters of mice administered with GNP (E_1 and E_2) or with the vehicle (PBS), after i.v. administration. The time points studied were: 24h after i.v. administration for GNP (E_1) and 48h after i.v. administration for GNP (E_2). The animals administered with vehicle were analyzed both at 24h and 48h after i.v. administration and the values obtained were pooled. Values were obtained directly from a Spotchem EZ equipment at Vall d’Hebron Research Institute or from a specific clinical veterinary service (“Servei de Bioquímica Clínica Veterinària” at the Universitat Autònoma de Barcelona). Results indicate that renal and hepatic function is maintained inaltered in GNP administered mice.

	n	BUN (mg/dL)		CREA (mg/dL		TP (g/dL)		ALB (g/dL)		AST (UI/L)		ALT (UI/L)		BIL (mg/dL)		GLU (mg/dL)	
		Mean	SEM	Mean	SEM	Mean	SEM	Mean	SEM	Mean	SEM	Mean	SEM	Mean	SEM	Mean	SEM
**GNP (E_1)**	3	79.3	4.6	0.31	0.01	4.52	0.09	2.33	0.03	80.3	14	19.3	2.4	0.05	0.01	190	28
**GNP (E_2)**	3	43.2	5.4	0.33	0.01	3.60	0.24	2.50	0.06	148	56	26.0	8.1	0.03	0.02	273	17
**Vehicle**	7	52.6	7.2	0.32	0.01	3.95	0.21	2.46	0.06	148	35	26.9	2.7	0.05	0.01	218	27

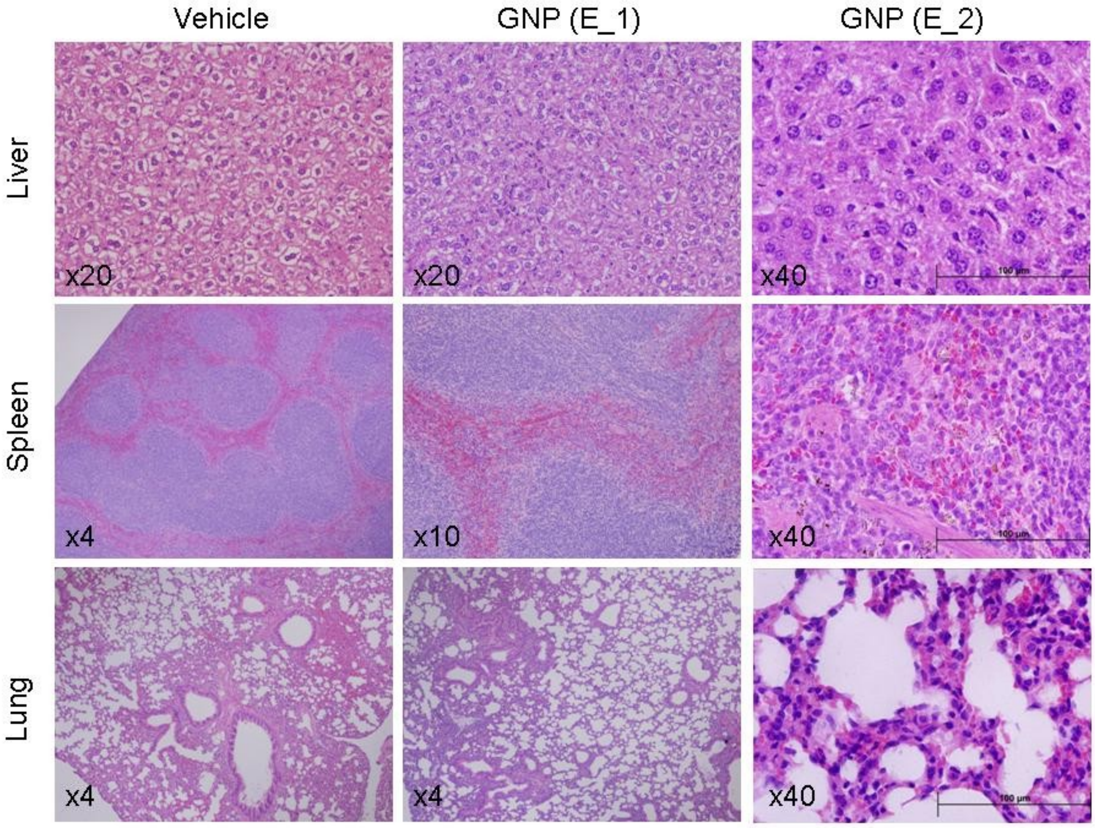

Supplementary Figure 2: Haematoxilin-Eosin stained sections of mice administered with PBS, GNP (E_1) (both 14 days after i.v. administration and GNP (E_2) (24h after i.v. administration). Images of tissues from GNP (E_2) administered animals shown in the last column were acquired with x40 magnification, whereas sections of vehicle or GNP (E_1) administration correspond to x10 (liver) or x4 (spleen an lungs) magnifications. All images were acquired with a BX61 Olympus microscope..

2. Application of the *ex vivo* postmortem method to iron oxide nanoparticles

Methods

Superparamagnetic iron oxide nanoparticle (SPION) synthesis:

The negative contrast agents evaluated in this work consisted in different *TEG*- or DMSA-coated SPIONs. Water-dispersible TEG-coated SPIONs were produced through a synthesis pathway described by Cai and Wan [2], with slight modifications [3]. In these experiments, a mixture of iron acetyl acetonate [Fe(acac)$_3$] and triethylene glycol was heated at 180ºC, leading to the partial decomposition of the reactants and the formation of an intermediate alkoxy-acetylacetonate-Fe^{3+}. After that, the heating of this mixture at 280ºC produced the reduction and subsequent decomposition of these complexes leading to the nucleation and final growth of the iron oxide nanoparticles. The resulting particles were washed with a mixture of ethyl acetate and ethanol, collected with the help of a magnet and transferred to a phosphate buffered saline (PBS) solution. This so-obtained colloidal dispersion presented particle size homogeneity (~ 5 nm) and good particle size distribution, with a mean hydrodynamic aggregate of 16 nm measured using dynamic light scattering (DLS) in a 90 Plus apparatus (Brookhaven). DMSA-coated SPIONs were obtained by replacing TEG coating for DMSA molecules, using a ligand-exchange reaction process described previously [4]. In this process, the

DMSA molecules attach to the SPIONs surface through carboxylic chelate (-COOH) bonding. The carboxylic acid groups (-COOH) of the DMSA molecules react with the Fe–OH bond at the surface of iron oxide nanoparticles via an acid-base reaction, giving Fe–O–C bonds with elimination of the H_2O; further stabilization of the ligand shells was achieved through intermolecular disulfide cross-linkages between the thiol groups (-SH) of the DMSA molecules, producing a well dispersed suspension of DMSA-coated iron oxide nanoparticles, showing a mean hydrodynamic aggregate size of 30 nm and excellent long-term stability. Chemicals were purchased from Sigma-Aldrich.

In vitro **relaxivity studies**

1.4 T studies

Five different dissolutions were prepared in NaCl 0.9% (B.Braun, Melsungen, Germany), containing between 0 and 1 mM of Fe each, prepared from stock solutions (Sinerem 21mg/mL, TEG 0.91mg/mL, DMSA 0.72mg/mL) with Fe content previously measured by ICP-MS with a PERKIN ELMER, ELAN 6,000 Spectrometer. The T2 measurements were carried out at 37 ºC in a Bruker Minispec MQ-60 NMR spectrometer (Bruker Optik, Ettlingen, Germany) at 1.41 T operating frequency at INA, Zaragoza, Spain.

Relaxivity r_2 was obtained from the slope of the curve $1/T_2$ vs the concentration of Fe expressed in mM.

7.0 T Studies

For the SPION studies, plastic tubes (3 mm internal diameter) were used, placed in polyethylene foam. Four different solutions ranging from 0.08 to 0.64 mM in Fe were prepared from the same stock solutions described for 1.4T studies, and used for MR scanning.

All 7 T studies were carried out at the joint NMR facility of the Universitat Autònoma de Barcelona and CIBER-BBN (Cerdanyola del Vallès, Spain), essentially with the acquisition parameters described in the main text in the "*in vitro* relaxivity studies- 7.0T" section.

TEG/DMSA SPIONs relaxivity studies

For T_2 maps acquisition, a MSME sequence was used; FOV, 30x45 mm; MTX, 256x128 (117x352 μm/pixel); NS, 3; ST, 2 mm; TR, 5000 ms; TEs were according to the following list: 8.5, 17, 25.5, 34, 42.50, 51, 59.5, 68.0, 76.5, 85.0, 93.5, 102.0, 110.5, 119.0, 127.5, 136.0, 144.5, 153.0, 161.5, 170.0, 178.5, 187.0, 195.5, 204.0, 212.5, 221.0, 229.5, 238, 246.5 and 255 ms; NA, 1; TAT, 8 min 10 sec.

***Ex vivo* post-mortem MRI Studies**

The CAs to be used for post mortem *ex vivo* evaluation were dissolved in saline solution (0.9% NaCl, B.Braun) in order to achieve solutions of comparable concentrations of the desired metal. The amount finally used for each animal was 8.2 ng of Fe dissolved in 4 μl of saline solution, prepared from the same stock solutions described for 1.4T in vitro studies. A commercial solution of an iron oxide-based CA (Ferumoxtran-10, SINEREM® Guerbet, Roissy, France) was used as a standard for SPIONs.

A T_2-weighted image acquisition was performed using the same axial coronal sections described previously in "*in vitro* 7 T studies" in the main text.

For this, a RARE sequence was used with: RARE factor, 8; FOV 17.6x 17.6 mm; MTX, 256x256 matrix (69x69 µm/pixel); TR/TE, 4000/12 ms; ST, 1mm; NA, 1; NR, 1; TAT, 3 min 12 sec. In both cases, the animals were anesthetized and handled as described for "*in vivo* studies". After that, animals were sacrificed and contrast administered as described in the "*ex vivo* post-mortem studies" section in the main text. After this, the T_2 weighted image acquisition was repeated as above.

Processing and post-processing of MR data

The processing and post-processing or MR data were done essentially as described in the main text. The only remarkable difference is that for negative contrast agents, all slices with noticeable RCE effect were taken into account for RCE calculation.

Results

Iv vitro results

The r_2 relaxivity values for the negative CAs were measured at 1.4 T and 7.0 T, and are listed in the Supplementary Table 3. The value obtained for Ferumoxtran-10 (iron nanoparticle compound currently commercialized as a negative CA) at 1.4 T *in vitro* is in good agreement with literature values also obtained *in vitro* (65 $mM^{-1}s^{-1}$) at the same temperature [5]. The variation in the r_2 relaxivity values obtained for SPIONs samples with respect to the value

measured for Ferumoxtran-10 may be explained by the variation on the mean hydrodynamic aggregate size. The triethylene glycol (TEG, SPION 1) (15 nm) and dimercaptosuccinic acid (DMSA, SPION 2) (30 nm) samples have different aggregate size in comparison to the size reported in the literature for Ferumoxtran-10 (20 nm) [6]. As expected, the increase of the SPIONs aggregate size markedly increases r_2 at 1.4 T from 48.0 to 119.6 $mM^{-1}s^{-1}$, which is coincident with the results previously reported by Muller and collaborators [7] related to the variation of the relaxivity of superparamagnetic iron oxide MRI contrast agents in function of their aggregate size.

Ex vivo postmortem results

A representative T_2-weighted image for SPIONs, and typical ROIs selected for analysis, are shown in Figure 3, A and B. Slight but significant differences in contrast were detected between the performance of the DMSA-coated SPIONS and Ferumoxtran-10 (Supplementary Figure 3C and Supplementary Table 2). This was not observed for TEG-coated SPIONS, possibly due to the higher variability observed for their values.

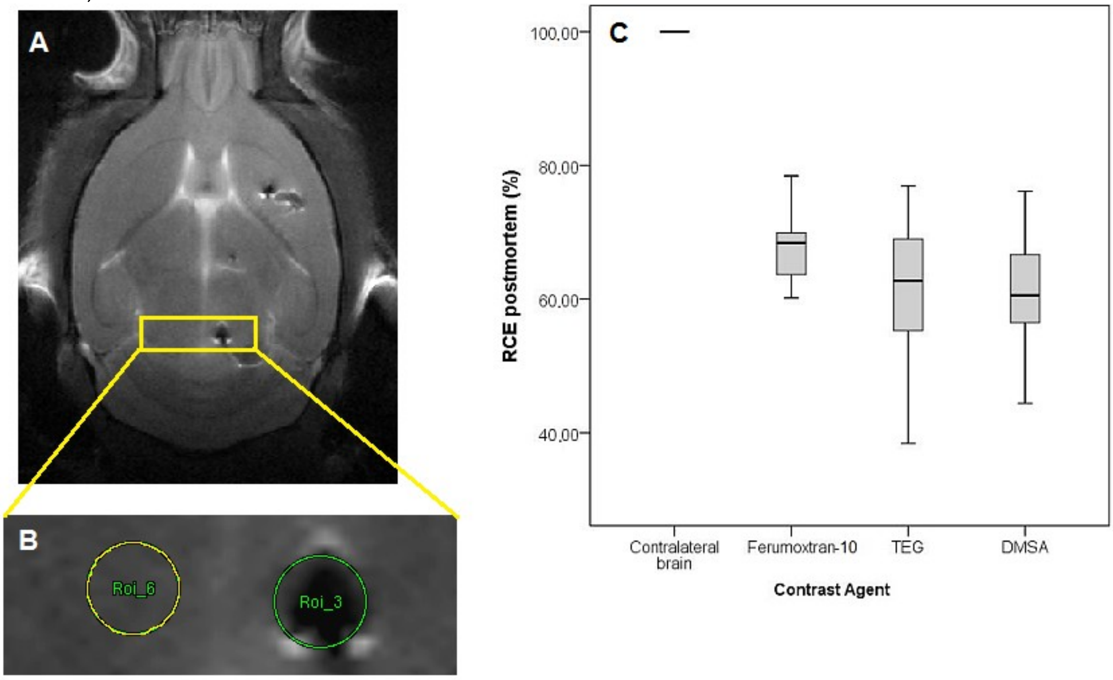

Supplementary Figure 3. A) Representative T_2-weighted image and example of ROIs used (manually drawn, green and yellow lines) for signal enhancement calculation for DMSA (SPION 2). B) Enlarged bottom row (white discontinuous rectangle), C) Boxplot of relative contrast enhancement for contralateral brain, DMSA, TEG and Ferumoxtran-10 obtained from ROIs of SPIONS studied. RCE was calculated using equation 1 given in the main text.

Supplementary Table 2: SPIONs studied, relaxivity (r_2) values at 1.4T and 7T, and *ex vivo* RCE

**Contrast agent**	**1.4T r_2 relaxivity ($s^{-1}mM^{-1}$) (*in vitro*) (n=3)**	**7.0T r_2 relaxivity ($s^{-1}mM^{-1}$) (*in vitro*) (n=3)**	**% RCE (*ex vivo*) (n=3)**
Ferumoxtran-10	66.1 ± 0.3	63.5±16.2	-31.8 ± 5.4
TEG (SPION1)	48.0±2.1	67.7±4.2	-39.3 ± 11.0
DMSA (SPION2)	119.6±2.5***	15.2±2.6**	-38.7 ± 8.2*

* p<0.05 versus Ferumoxtran-10
**p<0.05 versus TEG and Ferumoxtran-10
***p<0.05 versus TEG
For each value mean and standard deviation are shown

Discussion

The negative contrast agents showed differences between the r_2 *in vitro* relaxivity values (*in vitro*) and the RCE changes *(ex vivo)*. Thus, the *ex vivo* performances of DMSA and TEG (Supplementary Figure 3 and Supplementary Table 2) are closer to that of the commercial agent Ferumoxtran-10 than what could be expected from *in vitro* measurements. This suggests the possible interest of further evaluating the post mortem *ex vivo* protocol for negative candidate CA performance assessment prior to advancing towards all additional stages of *in vivo* performance analysis.

Reference list for supplementary data

1. Marradi M, Alcántara D, de la Fuente J, García-Martín M, Cerdan S, Penadés S (2009) Paramagnetic Gd-based gold glyconanoparticles as probes for MRI: tuning relaxivities with sugars. Chem Commun 26:3922-3924
2. Cai W, Wan J (2007) Facile synthesis of superparamagnetic magnetite nanoparticles in liquid polyols. J Colloid Interface Sci 305 (2):366-370
3. Miguel-Sancho N, Bomatí-Miguel O, Colom G, Salvador J-P, Marco M-P, Santamaría J (2011) Development of Stable, Water-Dispersible, and Biofunctionalizable Superparamagnetic Iron Oxide Nanoparticles. Chem mater 23:2795-2802
4. Roca AG, Carmona D, Miguel-Sancho N, Bomatí-Miguel O, Balas F, Piquer C, Santamaria J (2012) Surface functionalization for tailoring the aggregation and magnetic behaviour of silica-coated iron oxide nanostructures. Nanotechnology 23 (15):155603
5. Corot C, Robert P, Idée JM, Port M (2006) Recent advances in iron oxide nanocrystal technology for medical imaging. Adv Drug Deliv Rev 58 (14):1471-1504
6. Laurent S, Forge D, Port M, Roch A, Robic C, Vander Elst L, Muller RN (2008) Magnetic iron oxide nanoparticles: synthesis, stabilization, vectorization, physicochemical characterizations, and biological applications. Chem Rev 108 (6):2064-2110
7. Muller RN, Elst LV, Roch A, Peter JA, Csajbok E, Gillis P, Gossuin Y (2005) Relaxation by metal-containing nanosystems. Adv Inorg Chem 57:239-292
